# Comparison Between Endobronchial‐Guided Transbronchial Biopsy and Computed Tomography–Guided Transthoracic Lung Biopsy for the Diagnosis of Central Pulmonary Lesions

**DOI:** 10.1111/crj.70015

**Published:** 2024-09-24

**Authors:** Cheng Zhang, Senlin Zhu, Yanliang Yuan, Shenhui Dai

**Affiliations:** ^1^ Thoracic Surgery Department The Affiliated Hospital of Xuzhou Medical University Xuzhou Jiangsu China; ^2^ Cardiothoracic Surgery Department The First Affiliated Hospital of Anhui University of Science & Technology Huainan Anhui China

**Keywords:** biopsy, central pulmonary lesions (CPLs), computed tomography–guided percutaneous transthoracic needle biopsy (PTNB), electronic bronchoscopy–guided transbronchial lung biopsy (TBLB), pathological diagnosis

## Abstract

**Background:**

Lung cancer is one of the most common malignant tumors at present. This study aimed to compare the diagnostic accuracy, complication rates, and predictive values of computed tomography (CT)–guided percutaneous transthoracic needle biopsy (PTNB) and electronic bronchoscopy–guided transbronchial lung biopsy (TBLB) for patients with central pulmonary lesions (CPLs) with a diameter ≥ 3 cm.

**Methods:**

We retrospectively included 110 patients with CPLs with a diameter ≥ 3 cm who underwent preoperative PTNB and TBLB examinations and ultimately underwent surgery to remove CPLs and obtained pathological results. Detailed information was collected in order to compare whether there was a difference between two groups. Data were processed using SPSS software (Version 26.0; IBM Corp). Data were compared by *t*‐test or chi‐square test. *p* < 0.05 was considered statistically significant.

**Results:**

All patients underwent surgical treatment at the department of thoracic surgery and obtained a final pathological diagnosis. The rate of positive predictive value (PPV) was comparable between the two methods, and the negative predictive value (NPV) was significantly higher in the PTNB group compared with the TBLB group (*p* < 0.05). In addition, PTNB was more sensitive and accurate than TBLB (*p* < 0.05). However, the PTNB group had a higher probability of complications, and TBLB was a relatively safer examination method.

**Conclusion:**

PTNB demonstrated a higher accuracy and sensitivity than TBLB in the treatment of CPLs with a diameter ≥ 3 cm, but the complication rates of PTNB are relatively high. These methods exhibited different diagnostic accuracies and therefore should be selected based on different medical conditions.

## Graphical abstract

We compared two biopsy methods for pulmonary lesions, computed tomography (CT)–guided percutaneous transthoracic needle biopsy (PTNB), and endobronchial‐guided transbronchial lung biopsy (TBLB). As a result, the rate of positive predictive value was comparable between the two methods, and the negative predictive value was significantly higher in the PTNB group compared with TBLB group. Besides, the PTNB group was also more sensitive and accurate than the TBLB group. However, PTNB had a higher probability of complications, and TBLB was relatively a safer examination method.

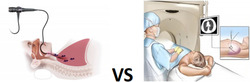

## 1 Introduction

Lung cancer poses a serious threat to human health; however, owing to the complexity of the pathogenesis of lung cancer, there are currently no effective drugs or methods to treat patients with lung cancer [1, 2, 3]. Therefore, a safe and accurate diagnosis of the pulmonary lesions is crucial. Nowadays, there are several advanced guidance systems such as fluoroscopy and electromagnetic navigation bronchoscopy to obtain pathological results. However, for some grassroots hospitals in China, limited by funding and medical equipment, traditional examination methods are still being carried out. The percutaneous transthoracic needle biopsy (PTNB) and transbronchial lung biopsy (TBLB) are the two commonly used methods to diagnose central pulmonary lesions (CPLs) [4, 5]. PTNB is primarily used for peripheral pulmonary lesions, and TBLB is performed for CPLs. These methods have their advantages and limitations. However, in the case of a relatively bigger CPL, both methods can be used. The choice between PTNB and TBLB has always been controversial in clinical practice [6]. A plethora of studies have compared the diagnostic accuracy of PTNB and TBLB for pulmonary lesions [7]. PTNB is considered a minimally invasive examination method and can help physicians easily acquire pathological specimens, but its complications, including hemorrhage and pneumothorax, should not be ignored [8]. Therefore, special attention should be paid to these issues while planning a biopsy to avoid or minimize potential complications.

TBLB is used to detect central endobronchial or peribronchial lesions [9]. Most of the studies have indicated that CPLs with endobronchial involvement can be easily evaluated via a bronchoscopy [10]. Moreover, endobronchial biopsy is a better assessment to diagnose when tumors invade the trachea or bronchus.

To date, no reports have suggested which one is more suitable. Therefore, this study aimed to evaluate the diagnostic accuracy of PTNB and TBLB in patients with CPLs.

## 2 Patients and Methods

### 2.1 Patients

**FIGURE 1:**
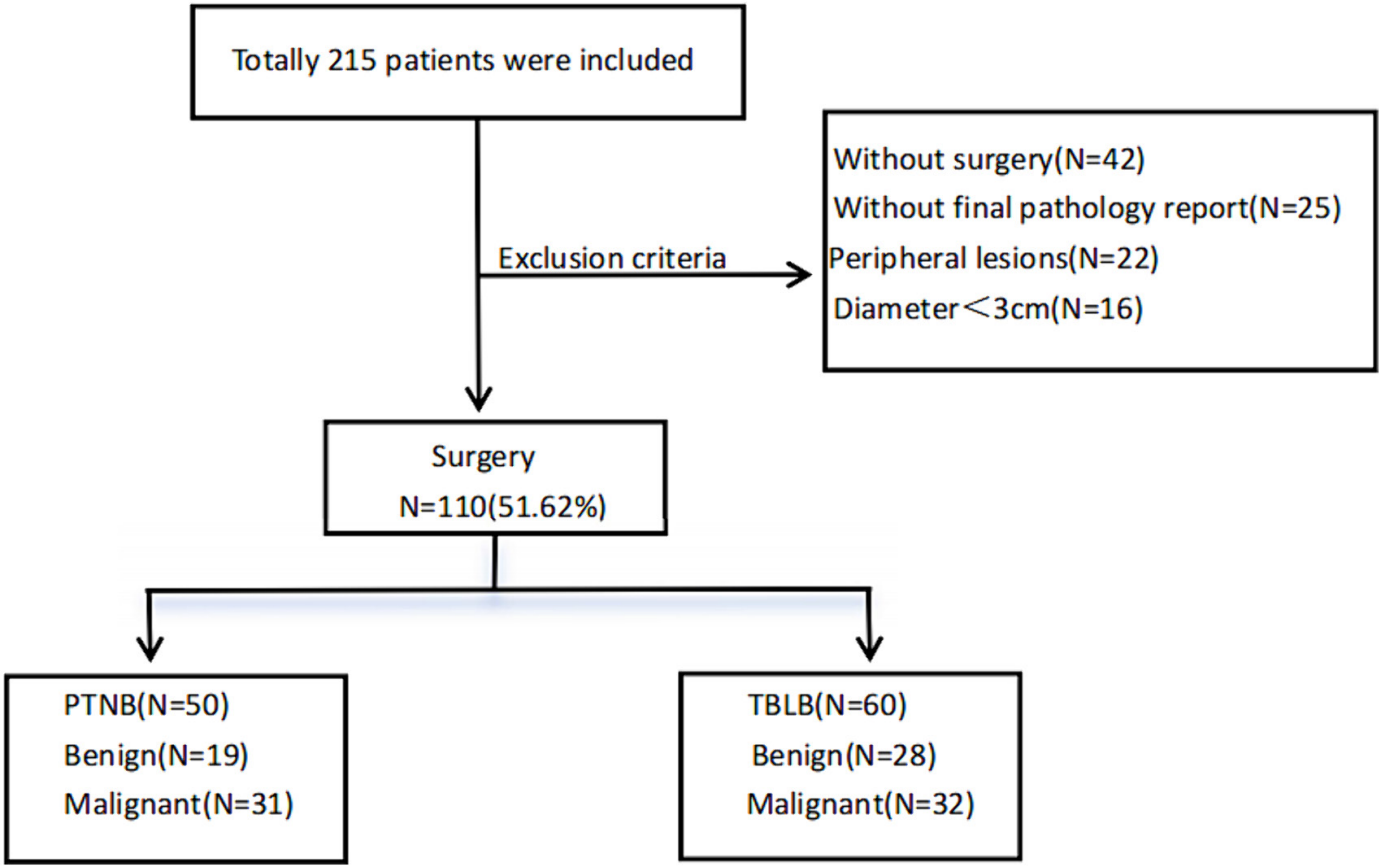
Inclusion and exclusion process for PTNB and TBLB groups.

This was a retrospective analysis study conducted from August 2020 to October 2022 comprising 110 patients at Xuzhou Medical University Affiliated Hospital, including 50 patients who underwent PTNB and 60 patients who underwent TBLB. All the patients underwent surgical treatment at the Department of Thoracic Surgery and obtained final pathological diagnosis. The study process and exclusion criteria are summarized in Figure 1.

### 2.2 TBLB and PTNB Techniques

#### 2.2.1 TBLB

According to the standard requirements of the “Diagnostic Flexible Bronchoscopy Application Guidelines (2018 Edition)” formulated by the Respiratory Disease Branch of the Chinese Medical Association [11], the patients were required to fast for 6–8 h, and the procedure was performed by using a traditional electronic bronchoscope (BF‐260, Olympus, Japan). After administering local anesthesia with 1% lidocaine (Cenexi, Italy), the patients were placed in a supine position, and the fiber‐optic bronchoscope was inserted through the nose. A 3–5 mL of diluted adrenaline solution (1 mg/10 mL sodium chloride solution) is infused into the bronchus. Then, the biopsy forceps are advanced to a predetermined depth according to the anatomical position of the lesion and then rotated. Once the forceps are properly positioned and rotated, they are opened to take a tissue sample when patients exhaled calmly. The tissue is then fixed in a 10% formalin solution and sent for pathological examination or smear examination. The process can be repeated two to three times if the patients can tolerate it.

#### 2.2.2 PTNB

Patients adopted a prone position or other suitable position based on the location of the lesions examined by computed tomography (CT; a low‐dose technique; slice thickness: 2 mm; Siemens, Germany). The best puncture position was selected to avoid pulmonary bullae, interlobular fissures, and other factors. A mark was made on the body surface. The patients were required to maintain a suitable position, and then the posture was fixed. The puncture angle and depth based on CT scan images were determined, and routine disinfection procedures were performed. Local anesthesia was administered by using 1% lidocaine, and the patients were required to hold their breath, and a Tru‐Cut 18‐G needle (Precisa, Italy) was quickly inserted to the predetermined depth. The patients were then required to breathe slowly, and then the correct position of the needle tip before inserting was determined by CT. Then, the patients were requested to hold their breath, and the needle core was quickly pulled out, with a visible size of approximately 1–3 mm tissue taken and sent for pathological examination. Biopsy could be repeated two to three times based on the quality of the specimen. The patients usually rested in the radiology department for about 30 min after surgery, and a chest x‐ray was performed to determine the presence of complications such as pneumothorax before shifting the patients to the ward.

### 2.3 Pathology Diagnosis

Histological and pathological types of pulmonary lesions were diagnosed based on the 2021 World Health Organization (WHO) Classification of Lung Cancer. All surgical specimens and corresponding biopsy specimens were diagnosed as malignant or benign and the histologic types were further confirmed. All surgical specimens were reassessed in consensus by two pathology experts (working experience > 10 years). The final diagnosis was reached by discussion in case of any disagreement. The pathological results obtained from the surgery are considered the gold standard.

### 2.4 Statistical Analysis

The data of the two groups are compared by *t*‐test. The enumeration data are compared by chi‐square test. *p* < 0.05 was considered statistically significant. SPSS software (Cersion 26.0; IBM Corp) was used for data processing.

## 3 Results

### 3.1 Comparison of the Basic Information of Patients Between TBLB and PTNB Groups

We conducted a retrospective analysis including 110 patients at Xuzhou Medical University Affiliated Hospital from August 2020 to October 2022. All patients underwent surgical treatment to remove CPLs, and obtained a final pathological diagnosis. The results summarized in Table 1. Of these, 52 were males, and 58 were females, aged between 27 and 80 years, with an average age of 58.74 (SD 11.82) years.

**TABLE 1:** The clinical information of patients with PTLB and BTNB.

	TBNB	PTLB	*p*
Gender			0.091
Male	24	28	
Female	36	22	
Age	61.08 ± 11.47	57.52 ± 10.26	0.392
BMI	21.11 ± 4.83	22.56 ± 6.31	0.054
Cardiovascular disease	8 (13.33)	12 (24.00)	0.152
Surgical anatomic site			0.423
Right upper lobe	28 (46.67)	21 (42.00)	0.512
Left upper lobe	6 (10.00)	9 (18.00)	
Right lower lobe	9 (15.00)	10 (20.00)	
Left lower lobe	9 (15.00)	7 (14.00)	
Right middle lobe	8 (13.33)	3 (6.00)	
Lesion size	4.07 ± 1.28	4.31 ± 1.46	0.362
Indicators			
CEA	3.14 ± 1.17	3.54 ± 1.28	0.089
CA125	10.81 ± 3.57	11.22 ± 2.41	0.492
NSE	13.96 ± 2.81	14.85 ± 2.28	0.068
Postoperative hospital stay	12.60 ± 1.74	9.09 ± 1.66	0.814

We first tested for the oncological blood indicators, such as carcinoembryonic antigen (CEA), cancer antigen 125 (CA125), and neuron‐specific enolase (NSE), and found no difference between the two groups. In addition, there was no statistically significant difference in gender, age, body mass index (BMI), tumor diameter, and locations of CPLs. The physical condition of patients between the two groups like cardiovascular disease was not comparable. Although there was no statistically significant difference in the length of the postoperative hospital stay between the two groups, the average postoperative hospital stay of the PTNB group was relatively short compared with the TBLB group (mean 12.60, SD 1.74 vs. mean 9.0, SD 1.66), which may be due to our limited sample size.

Based on the statistical results, 6% (3/50) of the patients reported pneumothorax and only one of these patients required thoracic closed drainage in the PTNB group, 6% (3/50) of the patients reported hemothorax, and 2% (1/50) of the patients had hemoptysis. These patients recovered through conservative treatment. In the TBLB group, 5% (3/60) of the patients experienced intratracheal bleeding, and all of them did not require special treatment. The complications that occurred in the two groups did not require any surgical intervention, and no fatal events were reported.

### 3.2 Comparison of the Diagnostic Rates Between TBLB and PTNB Groups

All patients underwent subpulmonary lobectomy for the lesions, and the pathological diagnosis was considered as gold standard. Table 2 summarizes the detailed results and the rate of malignancy (ROM) of the two groups based on the diagnostic criteria. We divided them into negative and positive categories based on the pathological results of the preoperative diagnosis. For the PTNB group, 18 patients were initially classified as benign tumors. Among them, two patients (11.11%) were confirmed to have malignant tumors by postoperative pathology, including one case of adenocarcinoma and one case of squamous cell carcinoma. Moreover, 32 patients were initially classified to have malignant tumors, but 1 patient was confirmed to have a benign lung tumor through surgical pathology.

**TABLE 2:** The number of malignant lesions and the ROM in TBLB and PTNB groups.

	Test‐negative					Test‐positive				
	*N*	Malignancy	ROM (%)	X^2^	*p*	*N*	Malignancy	ROM (%)	X^2^	*p*
TBLB	26	12	46.15	4.51	0.032	34	32	94.12	0.00	1.000
PTNB	18	2	11.11			32	31	96.88		

In contrast, for the TBLB group, of 34 patients who were previously diagnosed as positive samples, two (5.88%) were ultimately diagnosed to have benign tumors, including one adenomatous hyperplasia and one bronchial adenoma. There were 12 (46.15%) cases of postoperative misdiagnosed negative malignant tumors in the TBLB group, all of which were confirmed to be squamous cell carcinoma and adenocarcinoma based on the final surgical pathology. The results summarized in Table 3 revealed that the accuracy of the PTNB group (94.00%) was higher than that of the TBLB group (74.67%), and there was no statistically significant difference in the positive predictive value (PPV) between the two groups. The sensitivity and negative predictive value (NPV) rates were relatively higher in the PTNB group, and the difference was statistically significant. According to the final diagnosis, the malignant diagnosis rates of the two groups were equivalent.

**TABLE 3:** Comparison of diagnostic performance between TBLB and PTNB groups.

	PTNB	TBLB	X^2^	*p*
Sensitivity (%)	93.94	72.73	4.37	0.042
Specificity (%)	94.12	87.50	0.44	0.513
Accuracy (%)	94.00	74.67	5.02	0.029
Positive predictive value (%)	96.88	94.12	0.29	0.592
Negative predictive value (%)	88.89	53.85	4.51	0.034

### 3.3 Comparison of the Complications Between TBLB and PTNB Groups

Owing to the invasive nature of both examination methods, we conducted a statistical analysis of the incidence of complications in both groups. The results summarized in Table 4. In the TBLB group, three patients experienced hemoptysis during the examination, one of them underwent bronchoscopy again due to severe hemoptysis, and no significant complications were observed in other patients. The incidence rate of complications was 5%. Among 60 patients who underwent PTNB, seven patients experienced complications, with an incidence rate of 14%. Among them, three patients (5%) developed pneumothorax, three patients (5%) developed hemothorax, and one patient (1.67%) developed hemoptysis. Symptoms were alleviated through rest and symptomatic treatment. All the patients did not experience any pulmonary infections or life‐threatening situations such as shock during the examination. Of the two examination methods, the complications in the PTNB group were significantly higher than in the TBLB group, and the difference was statistically significant (*p* < 0.05).

**TABLE 4:** The incidence rates of complications between TBLB and PTNB groups.

Group	Hemoptysis	Pneumothorax	Hemothorax	Pneumonia	Total	*p*
PTNB (*n* = 50)	1	3	3	0	7(14%)	
TBLB (*n* = 60)	3	0	0	0	3(5%)	0.041

## 4 Discussion

In clinical practice, it is very important to diagnose pulmonary lesions at an early stage. At present, chest x‐ray and CT are minimally invasive procedures used to examine pulmonary lesions [12, 13]. However, these examinations can only elaborate on the imaging characteristics and cannot be used to provide conclusive diagnosis, especially in the case of diseases such as tuberculosis and tumors [14, 15]. Biopsy has become a method of choice in clinical practice, as it can quickly differentiate between the benign and malignant nature of pulmonary lesions [16, 17]. For peripheral pulmonary lesions, CT or ultrasound‐guided biopsy is usually performed. Bronchoscopy biopsy is usually used for CPLs. PTNB can be used when the lesions are large. Currently, physicians prefer PTNB and TBLB for pulmonary biopsy. In this study, 110 patients were divided into two groups to analyze the accuracy of PTNB and TBLB to diagnose CPLs with a diameter ≥ 3 cm. For TBLB, we can directly observe the mucosal surface and obtain histopathological indicators, making it more commonly used in clinical practice. However, PTNB can improve the positive detection rates of pulmonary lesions and accurately and effectively locate pulmonary lesions [18]. These methods demonstrate different diagnostic values in different clinical settings. The patients who participated in this study received surgical treatment and underwent pathological examinations to compare the diagnostic accuracy between the two groups. This study aimed to determine whether TBLB had any advantages over PTNB in clinical applications to avoid relying on subjective decisions made by radiologists or clinical physicians. However, there still exists a debate about the advantages and limitations of TBLB compared with PTNB in detecting CPLs, as there is no existing recommendation for their use [19, 20, 21]. According to the latest studies, the positive rates of TBLB in diagnosing CPLs can reach 70% [22, 23], whereas the positive rate of PTNB is 80% [24, 25], which is consistent with the results of the findings of this study. In this study, we found that TBLB had similar specificity in diagnosing lung tumors as PTNB [26, 27, 28]. However, the sensitivity and accuracy of PTNB in detecting malignant lesions were much higher than TBLB (sensitivity 93.94% vs. 72.73%, accuracy 94.00% vs. 74.67%). This is consistent with the findings of the previous studies, which indicate that the sensitivity and accuracy of PTNB in diagnosing malignant tumors are significantly higher than TBLB. In addition, we found no significant difference in the malignant diagnosis rates between the two groups. However, PTNB has relatively more incidence rates of complications, such as bleeding, pneumothorax, or some other complications consistent with other studies [29, 30, 31]. However, these complications were not fatal, and PTNB is a relatively safer examination method.

Our study has some limitations. First, data collection for this study was biased and did not compare lesions with smaller diameters, and samples without final pathology were excluded because surgical pathology results were considered as the gold standard. Second, there may be some factors that affect the statistical results, such as no more samples collected and possible biases among different pathologists in pathological diagnosis. However, we believe that these limitations had little impact on the results as our study addressed multiple steps. The next round of investigation should consider these issues.

In summary, our study demonstrated that the accuracy and sensitivity of PTNB are higher than TBLB; however, PTNB has increased complication rates in diagnosing pulmonary lesions. Both PTNB and TBLB demonstrated excellent performance and should be applied under the most suitable conditions to maximize their benefits.

## 5 Conclusion

This study indicated that PTNB demonstrated a higher accuracy and sensitivity than TBLB in diagnosing CPLs with a diameter ≥ 3 cm, but the complications of PTNB were relatively high. There was no statistically significant difference in the PPV between the two groups. The sensitivity and NPV values were relatively higher in the PTNB group, and the difference was statistically significant. Based on the final surgical diagnosis, the malignant diagnosis rates of the two groups were equivalent. These diagnostic modalities exhibited varied diagnostic performance and therefore should be adopted based on the specific medical conditions.

## Author Contributions

Yanliang Yuan designed and wrote the manuscript. Senlin Zhu and Shenhui Dai collected and analyzed the data. Cheng Zhang reviewed and edited the manuscript. All authors read and approved the final manuscript.

## Ethics Statement

This study was approved by the ethical committee of Affiliated Hospital of Xuzhou Medical University. All authors have approved the submission and publishment of this manuscript.

## Conflicts of Interest

The authors declare no conflicts of interest.

## Data Availability

Data availability on request from the author.
